# PRRSV-Vaccinated, Seronegative Sows and Maternally Derived Antibodies (II): Impact on PRRSV-1 Vaccine Effectiveness and Challenge Outcomes in Piglets

**DOI:** 10.3390/vaccines12030257

**Published:** 2024-03-01

**Authors:** Jorian Fiers, Dominiek Maes, Ann-Brigitte Cay, Frank Vandenbussche, Laurent Mostin, Anna Parys, Marylène Tignon

**Affiliations:** 1 Unit Viral Re-Emerging, Enzootic and Bee Diseases, Department Infectious Diseases in Animals, Sciensano, Groeselenbergstraat 99, 1180 Ukkel, Belgium; jorian.fiers@sciensano.be (J.F.); annbrigitte.cay@sciensano.be (A.-B.C.); frank.vandenbussche@sciensano.be (F.V.); 2 Unit of Porcine Health Management, Department of Reproduction, Obstetrics and Herd Health, Faculty of Veterinary Medicine, Ghent University, Salisburylaan 133, 9820 Merelbeke, Belgium; dominiek.maes@ugent.be; 3 Unit Experimental Centre, Department Infectious Diseases in Animals, Sciensano, Kerklaan 68, 1830 Machelen, Belgium; laurent.mostin@sciensano.be (L.M.); anna.parys@sciensano.be (A.P.)

**Keywords:** PRRSV, vaccination, viremia, challenge, immune response, maternally derived antibodies

## Abstract

Vaccination against the Porcine Reproductive and Respiratory Syndrome virus (PRRSV) is widely practiced in both sows and piglets. However, it has been shown that multivaccinated sows sometimes lack a detectable antibody response, testing seronegative in ELISA (non-responders). Moreover, PRRSV-vaccinated piglets can remain seronegative as well, which is mainly attributed to the interference of maternally derived antibodies (MDAs). The current study investigated the impact of the sow’s immune status on the PRRSV vaccine effectiveness in the progeny. The experimental trial included forty-eight piglets (*n* = 48) originating from a commercial Belgian breeding herd, with twenty-four piglets born from PRRSV vaccinated responder sows (E+ piglets) and twenty-four piglets born from PRRSV vaccinated non-responder sows (E− piglets). Eight piglets in each group were either non-vaccinated (NoVac piglets; *n* = 8), intramuscularly vaccinated (IM piglets; *n* = 8), or intradermally vaccinated (ID piglets; *n* = 8), with the same PRRSV-1 vaccine as used in the sow population. Vaccination was performed at weaning at three weeks of age, and all study piglets were challenged with a high dose of the PRRSV-1 07V063 strain at 6 weeks of age. A clear interference of MDAs was observed in the E+ piglets: 66.7% of the vaccinated E+ piglets lacked an antibody response at 3 weeks post-vaccination (non-responders). Consequently, post-challenge, only the responding E+ piglets had a significantly reduced serum viremia compared to the E+ NoVac piglets. The observed viremia in the non-responding E+ piglets was similar to the viremia of the E+ NoVac piglets. In the vaccinated E− piglets, a lack of antibody response at 3 weeks post-vaccination was observed in 18.8% of the piglets. Interestingly, despite the lack of a vaccine antibody response, the non-responding E− piglets had a significantly reduced serum viremia compared to the NoVac E− piglets. In contrast, the viremia of the responding E− piglets was only numerically reduced compared to the NoVac E− piglets. Finally, some clear differences were observed in both the kinetics of infection and the immune responses post-challenge between the E+ and E− piglets. The results of this study confirm the consequences of the MDA interference on the induced partial protection of PRRSV vaccination in experimentally challenged piglets. More research is warranted to understand the immunological mechanisms behind MDA interference in PRRSV vaccination and to explain the observed differences between E+ and E− piglets.

## 1. Introduction

Porcine Reproductive and Respiratory Syndrome (PRRS) is one of the most challenging diseases in pig production worldwide. More than three decades ago, the disease was almost simultaneously described in the USA and in Europe [1,2,3]. The infectious pathogen causing the disease is a small RNA virus belonging to the order *Nidovirales*, family *Arteriviridae:* the PRRS-virus (PRRSV). This virus is nowadays classified as two different species: the *Betaarterivirus suid 1* and *Betaarterivirus suid 2*, sharing nucleotide similarity of approximately 55% [4,5,6,7]. The former is better known as PRRSV-1 and is the dominant species in Europe, while the latter is known as PRRSV-2 and is mainly present in North America. Despite mass vaccination being practiced, the disease is still responsible for enormous production and economic losses worldwide [8,9]. The main disease manifestations include reproductive dysfunction in sows and gilts (abortions, the birth of weak and/or dead piglets, irregular return to estrus) and respiratory distress in piglets and fattening pigs (coughing, sneezing, dyspnea, growth retardation). Additionally, PRRSV infection leads to a reduction in respiratory immunity, making PRRSV-infected pigs more susceptible to secondary infections [10]. 

PRRSV vaccination is widely practiced and can have beneficial effects on the clinical manifestation of the disease and transmission of the virus [11,12,13]. However, vaccination does not lead to complete protection against infection, and several factors are responsible for the suboptimal effectiveness of the currently available PRRSV vaccines [14,15,16]. First, PRRSV is known to have a high genetic diversity, which is caused by both random mutations due to the lack of proofreading by the PRRSV RNA polymerase and recombination events between different PRRSV strains. Consequently, both immune escape mutants and highly virulent strains are present in the field [17,18,19,20,21]. Second, several studies have shown that PRRSV modulates the immune response. Both an inhibition of innate immunity and poor adaptive immune responses have been described. Mainly, the limited production of neutralizing antibodies and the low induction of PRRSV-specific interferon-γ secreting cells are responsible for the lack of protective immunity [22,23,24,25]. Finally, it has been shown that a proportion of PRRSV-vaccinated sows and/or piglets lack a PRRSV-specific antibody response, with vaccinated pigs remaining seronegative in commercial ELISA kits [26,27,28,29,30,31,32]. In multivaccinated sows, the origin of this non-responsiveness upon vaccination remains unknown and warrants further investigation [26]. Recently, our research group has investigated the consequence of this seronegative sow status for their progeny by experimentally challenging piglets born from both PRRSV seropositive and PRRSV seronegative sows that were routinely PRRSV vaccinated and originated from a commercial Belgian breeding herd [33]. The lack of maternally-derived antibodies (MDAs) in piglets born from the non-responding sows resulted in a higher viral load and increased nasal shedding in the first days following the experimental challenge, compared to piglets born from the seropositive sows, who had received MDAs from their mother. In vaccinated piglets, the origin of vaccine non-responsiveness is better understood and related to the presence of MDAs, which interfere with the vaccine response. In intramuscular vaccinated piglets, the MDA interference has been described by different research groups [27,28,29]. However, the immunological mechanisms behind MDA interference and the consequences of MDA interference on vaccine effectiveness after infection remain understudied. 

The current study aimed to assess the PRRSV vaccine effectiveness against an experimental PRRSV-1 challenge in piglets born from PRRSV-vaccinated seropositive (responding) sows and piglets born from PRRSV-vaccinated seronegative (non-responding) sows. It could be hypothesized that piglets born from the non-responding sows show better vaccine effectiveness because they lack MDAs and are thus not susceptible to MDA interference. However, since these piglets are born from sows with a disturbed vaccine response (no antibody production), it could also be hypothesized that the piglets will react less to the vaccine themselves. Finally, both groups of piglets were vaccinated either via the intramuscular route or via the intradermal route to investigate whether there was a difference between both routes in terms of MDA interference and vaccine effectiveness after the challenge. 

## 2. Materials and Methods

### 2.1. Study Design

**Figure 1:**
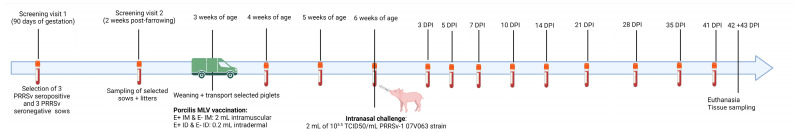
Overview of the experimental study design. Created using Biorender.com.

The Ethical Committee of Sciensano approved the study design, with approval number20221025-01. A schematic overview of the study design can be found in Figure 1. Forty-eight piglets (Hypor × German Piétrain), originating from a Belgian, PRRSV-stable, farrow-to-finish herd, were included in this study. The sows in the selected herd were routinely PRRSV-1 vaccinated in the following schedule: intramuscular Porcilis MLV (MSD, Rahway, NJ, USA) vaccination at 60 days of gestation and 6 days post-farrowing. Fifty-six breeding sows of mixed parity were blood sampled at 90 days of gestation for selection of three PRRSV-seropositive (responders to vaccination) and three PRRSV-seronegative (non-responders to vaccination) sows. At two weeks post-farrowing (wpf), the selected sows and ten piglets of each litter were blood sampled for analysis of PRRSV-specific and PCV2-specific antibodies. The latter was performed as a control for adequate colostrum intake, as previously described [27]. One week later, at 3 weeks of age (3 woa), twenty-four piglets originating from the three PRRSV-seropositive sows (E+ piglets) and twenty-four piglets originating from the three PRRSV-seronegative sows (E− piglets) were weaned and transported to the experimental facilities of Sciensano (Machelen, Belgium). The piglets were distributed in six experimental groups and each experimental group was housed in a separate compartment (Table 1): E+ NoVac (non-vaccinated E+ piglets; *n* = 8), E− NoVac (non-vaccinated E− piglets; *n* = 8), E+ IM (intramuscular vaccinated E+ piglets; *n* = 8), E+ ID (intradermal vaccinated E+ piglets; *n* = 8), E− IM (intramuscular vaccinated E− piglets; *n* = 8) and E− ID (intradermal vaccinated E− piglets; *n* = 8). Siblings were as much as possible equally distributed over the different experimental groups, ensuring that each experimental group consisted of eight piglets originating from three different sows. For example, eight siblings originating from E+ sow one were distributed as follows: three siblings in E+ NoVac, two siblings in E+ IM, and three siblings in E+ ID. Upon arrival, the E+ IM and E− IM groups were intramuscularly vaccinated in the right neck with 2 mL of the Porcilis MLV (MSD, Rahway, NJ, USA), while the E+ ID and E− ID groups were intradermally vaccinated with a hypodermic syringe in the right neck with the same dose of vaccine in a volume of 0.2 mL. Additionally, a Thermochip (MSD) was intramuscularly injected in the left neck of all piglets for measurement of body temperature throughout the study. A body temperature > 40 °C was considered to be fever. All piglets were sampled at 4 woa, 5 woa, and 6 woa to analyze the vaccine response. At 6 woa (3 weeks post-vaccination), all piglets were intranasally challenged with 2 mL containing 10^5.5^ tissue culture infectious dose with 50% end-point/mL (TCID_50_/mL) of the PRRSV-1 07V063 strain (1 mL/nostril). Challenge response was analyzed based on sampling at 3, 5, 7, 10, 14, 21, 28, 35, and 41 days post-infection (dpi). All serum samples were stored at −80 °C until analysis. Piglets were monitored daily for clinical signs. Half of the piglets were euthanized by electrocution and necropsied at 42 dpi, the other half at 43 dpi. Unfortunately, one E+ ID-challenged piglet died during blood sampling at 5 dpi; this piglet was excluded from the analysis. 

### 2.2. Viruses and Viral Titration

The strain used to intranasally challenge the piglets included in this study was a Belgian field isolate, the PRRSV-1 07V063 strain (GenBank: GU737264). This strain was isolated from a Belgian-infected herd in 2007 and was kindly provided by Dr. Hans Nauwynck (Ghent University) [34,35]. The viral strain was propagated in porcine alveolar macrophages (PAMs) for three passages, as previously described [33]. The challenge strain was diluted in sterile phosphate-buffered solution (Thermo Fisher Scientific, Waltham, MA, USA) to reach the viral titer of 10^5.5^ TCID_50_/mL used for intranasal inoculation. The PRRSV-1 DV strain was derived from the Porcilis MLV vaccine and cultured on MARC-145 cells as previously described [33]. The viral titer of the used challenge strain, as well as the viral titers of the serum samples collected at 3, 5, 7, 10, and 14 dpi, were determined by an in vitro infection assay. In short, a ten-fold dilution series was made of the samples to test, and these dilutions were used to infect cultivated PAMs. The infected PAMs were incubated for 72 h at 37 °C and 5% CO_2_. Following incubation, the cytopathogenic effect was assessed in each dilution, and the Reed-Muench method was used to calculate the final viral titer in each sample [36].

### 2.3. Antibody Analysis

Commercially available ELISA assays were used to determine the presence of both PRRSV-specific antibodies (Abs) and porcine circovirus type 2 (PCV2)-specific Abs. The former was assessed using the IDEXX PRRS X3 Ab test (IDEXX Laboratories, Westbrook, ME, USA), which is the most commonly used ELISA assay for PRRSV Ab testing [10]. The latter was assessed using the Biochek PCV2 Antibody Test (Biocheck, Reeuwijk, The Netherlands). The manufacturers’ guidelines were followed during the performance of both tests. Samples with a sample-to-positive S/*p* value ≥ 0.4 or a S/*p* value ≥ 0.5 were seropositive for PRRSV and PCV2, respectively. Finally, neutralizing antibodies (NAbs) against PRRSV were determined using a virus neutralization assay (VN), as previously described [27]. The NAbs were tested against 100 TCID_50_ of the PRRSV-1 DV strain (the used vaccine strain).

### 2.4. Cytokine Analysis

Commercially available ELISA assays (Invitrogen—Thermo Fisher Scientific, Waltham, MA, USA) were used to determine the post-challenge induction of cytokines. Firstly, the serum concentration (pg/mL) of interferon-gamma (IFN-γ) was assessed using the IFN gamma Porcine ELISA kit. Secondly, the tumor necrosis factor-alpha (TNF-α) concentration in serum was assessed using the TNF alpha Porcine ELISA kit. The manufacturers’ guidelines were followed in the execution of both tests. In each run, a standard dilution series with known IFN-γ or TNF-α concentration (provided in the kit) was included. GraphPad Prism 9 Software (GraphPad Software, San Diego, CA, USA) was used to generate the standard curve based on the concentration of the standard dilution series and the obtained optical density (OD) values of the standards, using a 4-point parameter algorithm. The OD values of the serum samples were then interpolated on the generated standard curve to obtain the concentration of serum IFN-γ and TNF-α in each sample. 

### 2.5. RT-qPCR

The IndiMag Pathogen kit and IndiMag 48 s instrument (Indical Bioscience, Leipzig, Germany) were used to extract RNA from the collected serum samples. Prior to the intranasal challenge, the presence of the MLV strain was determined using the VetMAX PRRSV EU and NA 2.0 kit (Thermo Fisher Scientific, Waltham, MA, USA) on the QuantStudio 5 Real-Time PCR System (Thermo Fisher Scientific, Waltham, MA, USA), to determine the MLV viremia. After the challenge, an in-house RT-qPCR was used to detect both the MLV and the challenge strain, using primers and probes specific for the PRRSV-1 DV strain and primers and probes specific for the PRRSV-1 07V063 strain (Integrated DNA Technologies, Coralville, IO, USA). During each extraction and RT-qPCR run, a standard curve was generated based on a four-point serial dilution series consisting of ten-fold dilutions of the PRRSV-1 07V063 strain. A relative quantification of the samples to test was performed based on this standard curve, as previously described [33]. 

### 2.6. Interferon-Gamma ELISpot

The commercially available ELISpot Plus: Porcine IFN-γ (ALP) kit (Mabtech AB, Nacka Strand, Sweden) was used to determine the number of IFN-γ secreting cells (IFN-γ SCs) specific for the PRRSV-1 DV strain. The PBMC isolation, stimulation, and ELISpot analysis were performed as previously described [33]. The CTL Q6 Ultra-V Analyzer (ImmunoSpot, Cleveland, OH, USA) was used for ELISpot plate read-out.

### 2.7. Statistics

The GraphPad Prism 9 software (GraphPad Software, San Diego, CA, USA) was used to perform statistical analysis and visualization of the results. The gathered data was first checked for normality using the built-in Sharpo-Wilk test. Statistical differences between experimental groups were calculated using the one-way ANOVA with Sidaks multiple comparison test or the Kruskal-Wallis test with Dunn’s multiple comparison tests for normal and non-normal distributed data, respectively. The area under the curve (AUC) values for fever and serum viremia were calculated using the built-in function of the GraphPad Prism 9 Software, with a baseline of Y = 0, ignoring peaks that are less than 0.1 Y units high and only taking into account peaks that go above the baseline. For the AUC for fever calculation, the measured temperature of each piglet at each time-point was first reduced by 40, and negative values (no fever) were given a value of zero. Finally, a comparison of AUC values between experimental groups was performed as described above. Throughout the article, results are written as mean ± standard deviation of the mean. Statistical tests with a *p*-value < 0.05 were considered statistically significant. Statistical significance is visualized with asterics on the graphs: * *p*-value < 0.05; ** *p*-value < 0.01; *** *p*-value < 0.005; **** *p*-value < 0.001.

## 3. Results

### 3.1. Sow and Piglet Selection

**Figure 2:**
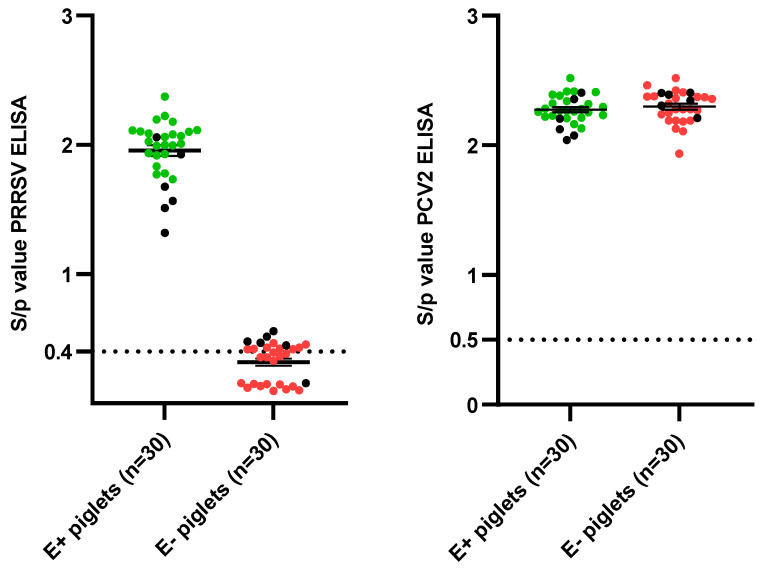
Overview of the presence of antibodies against PRRSV (**left**) and antibodies against PCV2 (**right**) in a selection of sixty piglets, originating from either routinely PRRSV vaccinated seropositive sows (E+ piglets; n = 30) or routinely PRRSV vaccinated seronegative or slightly seropositive sows (E− piglets; n = 30). In each group, twenty-four piglets were included in the experimental trial, represented by green (E+ piglets) or red (E− piglets) dots. The cut-off value for seropositivity in each ELISA test is visualized by a dotted line. The mean S/*p* values ± standard error of the mean (SEM) are presented by error bars.

Based on the sow sampling at 90 days of gestation, three responding sows (seropositive) and three non-responding sows (seronegative/slightly seropositive) to the routine PRRSV-vaccination were selected (Supplementary Figure S1A). A resampling of the selected sows was performed at 2 weeks post-farrowing (wpf) (Table 2). There was an absence of NAbs in all sows, except one E+ sow having a VN titer of 2 Log_2_ at 90 days of gestation. A clear presence of PRRSV-specific maternally-derived antibodies (MDAs) was observed in piglets born from the responding sows (E+ piglets), while these were absent in most of the piglets born from the non-responding sows (E− piglets) (Figure 2). None of the selected piglets had the presence of neutralizing MDAs. To ensure that the observed differences in the presence of PRRSV-specific MDAs were not due to differences in colostrum intake, the presence of PCV2-antibodies was analyzed in both the selected sows and piglets. All selected sows, both the three responding sows and the three non-responding sows, had a clear presence of PCV2-antibodies (Supplementary Figure S1B). Additionally, both the selected E+ piglets and the selected E− piglets had the presence of PCV2-specific MDAs, confirming an adequate colostrum intake in both groups (Figure 2).

### 3.2. Vaccine Responses

At three weeks of age (woa), the intramuscular or intradermal PRRSV MLV vaccination was performed. Clear differences in the vaccine responses were observed between the E+ vaccinated piglets and the E− vaccinated piglets. 

**Figure 3:**
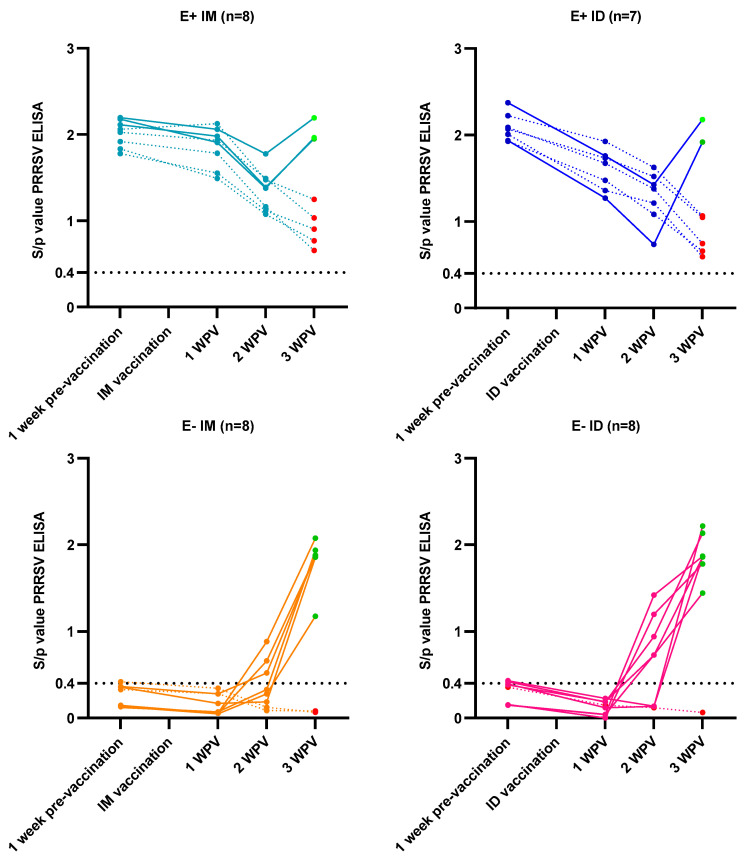
PRRSV antibody responses after intramuscular (IM) or intradermal (ID) MLV vaccination in 3-week-old piglets originating from either PRRSV vaccinated, seropositive sows (E+ piglets) or PRRSV vaccinated, seronegative/slightly seropositive sows (E− piglets). Results are shown as S/*p* values assessed by a commercial PRRS ELISA kit. The cut-off value of seropositivity (S/*p* ≥ 0.4) is indicated with a dotted line. Full connecting lines and green dots at 3 wpv represent piglets that showed an antibody response after PRRSV vaccination; dotted connecting lines and red dots at 3 wpv represent piglets without an antibody response after PRRSV vaccination.

The presence of MDAs interfered with the vaccine antibody response in the E+ vaccinated piglets, with a lack of antibody response observed in 5/8 (62.5%) and 5/7 (71.4%) E+ IM and E+ ID piglets, respectively, by 3 weeks post-vaccination (wpv) (Figure 3). In contrast, only 1 E− ID piglet (12.5%) and 2/8 (25%) E− IM piglets lacked a vaccine antibody response by 3 wpv (Figure 3). Additionally, the proportion of vaccinated piglets that tested PCR positive for the vaccine strain at 3 wpv was higher in the E− vaccinated piglets (E− IM: 6/8 and E− ID: 7/8) compared to the E+ vaccinated piglets (E+ IM: 5/8 and E+ ID: 2/7). Interestingly, the MLV strain was detected in one E+ IM and one E+ ID until 7 days post-infection.

**Figure 4:**
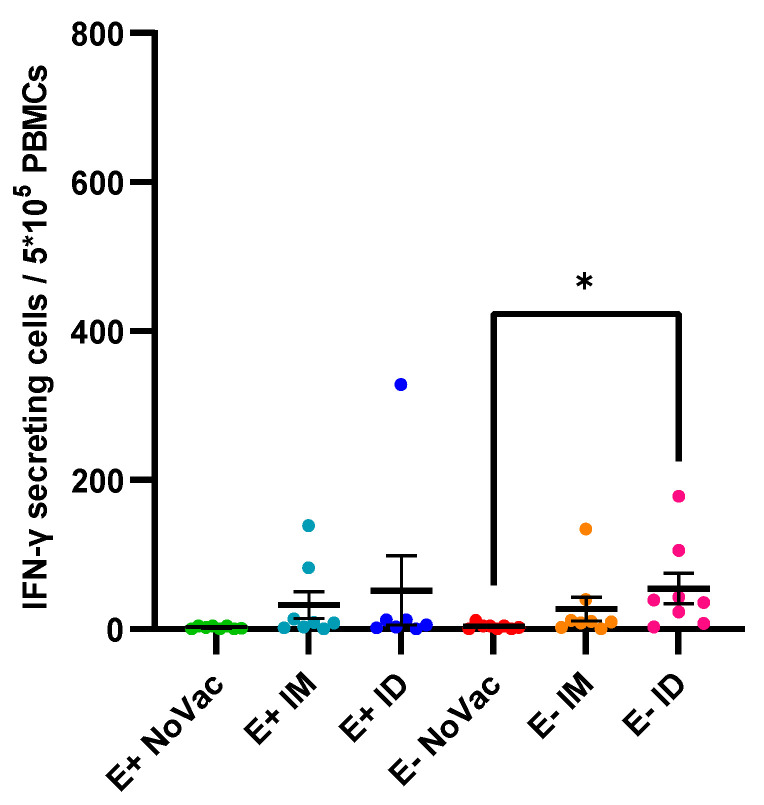
Overview of interferon-γ secreting cells (IFN-γ SCs) specific for the PRRSV-1 DV strain in piglets originating from routinely PRRSV vaccinated, seropositive sows (E+ piglets) or piglets originating from routinely PRRSV vaccinated, seronegative/slightly seropositive sows (E− piglets). Piglets were either non-vaccinated (NoVac), intramuscularly vaccinated (IM), or intradermally vaccinated (ID) at 3 weeks of age with the same PRRSV-1 MLV as used in the sows. The number of IFN-γ SCs was quantified at 3 weeks post-vaccination. Results are shown as dots for each piglet, with error bars representing the mean number of IFN-γ SCs ± standard error of the mean.

Next to the vaccine antibody responses, an analysis of the induction of PRRSV-specific IFN-γ secreting cells (IFN-γ SCs) at 3 wpv was performed. An induction of PRRSV-specific IFN-γ SCs was observed in a proportion of the vaccinated piglets but not in the non-vaccinated piglets (Figure 4). Two of the E+ IM piglets and one E+ ID piglet showed a high induction of IFN-γ SCs; two of these three piglets also had an antibody response by 3 wpv. In the other E+ vaccinated piglets (both responders and non-responders), only a limited induction of IFN-γ SCs was observed. Two of the E+ IM piglets had a relatively high number of IFN-γ SCs, while the presence of IFN-γ SCs was rather limited in the remaining six E+ IM piglets. Finally, the E− ID piglets (54.31 ± 59.09) had the highest overall number of IFN-γ SCs, which was significantly higher (*p* = 0.016) than the number of IFN-γ SCs in the non-vaccinated E− piglets (3.38 ± 3.39). 

### 3.3. Fever Induction

**Figure 5:**
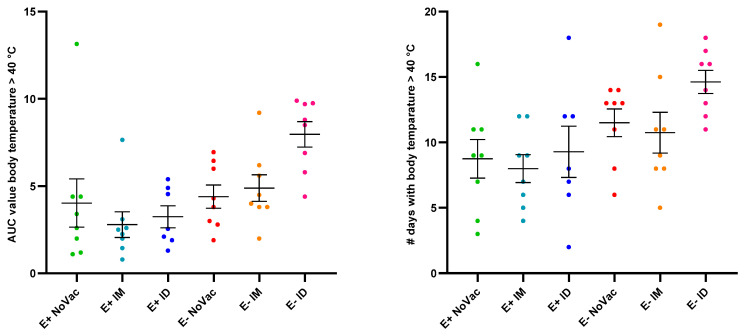
Overview of the induction of fever (body temperature > 40 °C) in piglets originating from routinely PRRSV vaccinated, seropositive sows (E+ piglets) or piglets originating from routinely PRRSV vaccinated, seronegative/slightly seropositive sows (E− piglets)Piglets were either non-vaccinated (NoVac), intramuscularly vaccinated (IM) or intradermally vaccinated (ID) at 3 weeks of age with the same PRRSV-1 MLV as used in the sows and all piglets were intranasally challenged with the PRRSV-1 07V063 strain at 6 weeks of age. Fever induction was determined from the day of infection to 24 days post-infection and visualized as the area under the curve (AUC) value for fever (**left**) or as the number of days with fever (**right**). The mean values ± standard error of the mean are shown as error bars for each experimental group.

Post-challenge, fever was observed from 2 dpi onwards in all experimental groups, and the body temperature of most piglets stabilized by 24 dpi (Supplementary Figure S2). Neither the area under the curve (AUC) value for fever, calculated from challenge day to 24 dpi, nor the number of fever days (until 24 dpi) were significantly different between experimental groups (Figure 5). However, a non-significantly higher induction of fever was observed in the E− piglets compared to the E+ piglets. Furthermore, the highest AUC value for fever and the highest number of fever days were observed in the E− ID piglets.

### 3.4. Serum Viral Load Quantified by RT-qPCR

**Figure 6:**
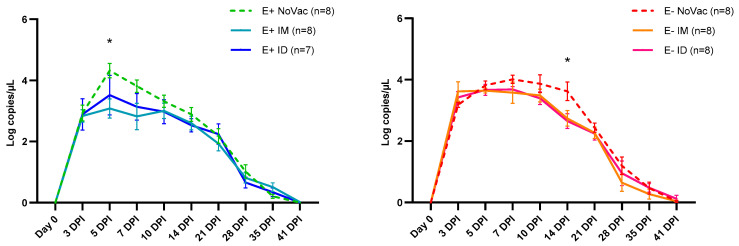
Kinetics of PRRSV-1 07V063 serum viral load, quantified by RT-qPCR, in piglets originating from routinely PRRSV vaccinated, seropositive sows (E+ piglets; **left**) or piglets originating from routinely PRRSV vaccinated, seronegative/slightly seropositive sows (E− piglets; **right**). Piglets were either non-vaccinated (NoVac), intramuscularly vaccinated (IM,) or intradermally vaccinated (ID) at 3 weeks of age with the same PRRSV-1 MLV as used in the sows, and all piglets were intranasally challenged with the PRRSV-1 07V063 strain at 6 weeks of age. The mean viral load ± standard error of the mean viral load is shown for each experimental group at each sampling moment.

The PRRSV-1 vaccination affected the serum viremia post-challenge, both in the E+ and E− piglets (Figure 6). At 3 dpi, there were no observed differences in the serum viral load between the non-vaccinated E+ piglets and the vaccinated E+ piglets (both IM and ID vaccinated). However, from 3 dpi until 14 dpi, serum viral load was reduced in both the E+ IM and E+ ID piglets compared to the E+ NoVac piglets. Furthermore, the viral load of the E+ IM piglets (3.08 ± 0.88 Log copies/µL) was significantly lower (*p* = 0.022) in comparison to the viral load of the E+ NoVac piglets (4.32 ± 0.66) at 5 dpi. Interestingly, a delayed effect of vaccination was observed in the E− piglets. In these piglets, the serum viral load remained similar between the E− NoVac piglets and both the E− IM and E− ID piglets until 7 dpi. Viral load was reduced in the vaccinated E− piglets (both E− IM and E− ID) compared to the non-vaccinated E− piglets in the period between 7 dpi and 14 dpi. At 14 dpi, the serum viral load was significantly lower (*p* = 0.029) in the E− ID piglets (2.65 ± 0.68 Log copies/µL) compared to the E− NoVac piglets (3.62 ± 0.86 Log copies/µL).

**Figure 7:**
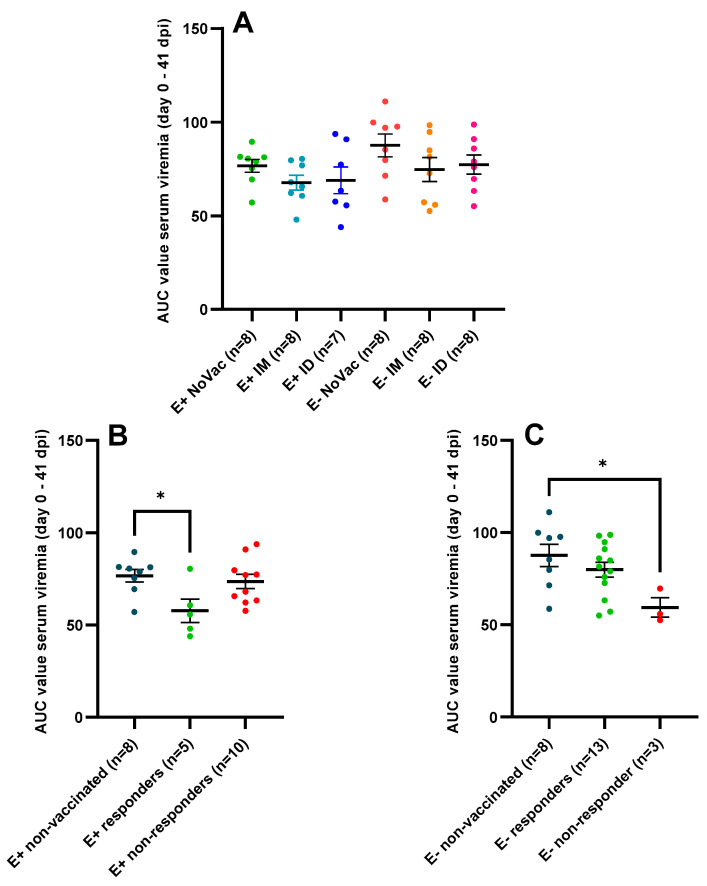
Serum PRRSV-1 07V063 viral load, quantified by RT-qPCR, in piglets originating from routinely PRRSV vaccinated, seropositive sows (E+ piglets), or piglets originating from routinely PRRSV vaccinated, seronegative/slightly seropositive sows (E− piglets). Piglets were either non-vaccinated (NoVac), intramuscular vaccinated (IM), or intradermal vaccinated (ID) at 3 weeks of age with the same PRRSV-1 MLV as used in the sows. All piglets were intranasally challenged with the PRRSV-1 07V063 strain at 6 weeks of age. Dots represent the area under the curve (AUC) value for serum viral load, which was calculated from the day of infection (day 0) to 41 days post-infection (dpi) for each piglet. The mean AUC value ± standard error of the mean AUC value are shown as error bars. (**A**) AUC values for the E+ NoVac, E+ IM, E+ ID, E− NoVac, E− IM, and E− ID piglets. (**B**) AUC values for the E+ subgroups based on the vaccine antibody response. E+ responder piglets had a vaccine antibody response by 3 weeks post-vaccination, while the E+ non-responder piglets lacked a vaccine antibody response by 3 weeks post-vaccination. (**C**) AUC values for the E− subgroups based on the vaccine antibody response at 3 weeks post-vaccination.

The lower serum viremia, observed in the vaccinated piglets compared to the non-vaccinated piglets, was confirmed by a comparison of the AUC values calculated from day 0 to 41 dpi (Figure 7A). A numerically higher AUC-value was observed in the E+ NoVac piglets (76.72 ± 9.77) compared to the E+ IM (67.71 ± 11.10) and E+ ID piglets (68.95 ± 18.83). Additionally, a numerically higher AUC-value was observed in the E− NoVac piglets (87.64 ± 17.13) compared to the E− IM (74.76 ± 18.00) and E− ID piglets (77.37 ± 14.52). The absence of a vaccine antibody response, which was observed in a large proportion of the E+ vaccinated piglets, influenced the overall serum viremia. A significantly lower (*p* = 0.033) and non-significantly lower (*p* = 0.073) AUC value was observed in the five E+ responders (57.75 ± 14.24), both IM and ID responders, compared to the AUC values observed in the eight E+ NoVac piglets (76.72 ± 9.77) and the ten E+ non-responders (73.55 ± 12.26) (Figure 7B). Conversely, the AUC values of the three E− non-responders (59.41 ± 9.09) were significantly lower (*p* = 0.035) and non-significantly lower (*p* = 0.13) than the AUC values of the eight E− NoVac piglets (87.64 ± 17.13) and the AUC values of the thirteen E− responders (79.91 ± 14.67) (Figure 7C).

### 3.5. Serum Viral Load Quantified by Viral Titration

The RT-qPCR analysis revealed a difference in the serum viral load between vaccinated and non-vaccinated piglets in the first two weeks post-challenge. To confirm these results and to assess the infectivity of the serum viral load until 14 dpi, an in vitro viral titration was performed. 

**Figure 8:**
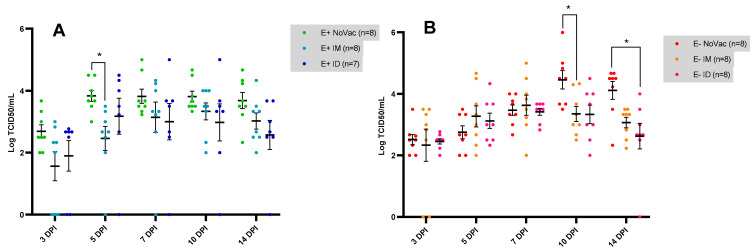
Serum viral load, quantified by in vitro viral titration, in piglets originating from routinely PRRSV vaccinated, seropositive sows (E+ piglets; (**A**)) or piglets originating from routinely PRRSV vaccinated, seronegative/slightly seropositive sows (E− piglets; (**B**)). Piglets were either non-vaccinated (NoVac), intramuscularly vaccinated (IM), or intradermally vaccinated (ID) at 3 weeks of age with the same PRRSV-1 MLV as used in the sows, and all piglets were intranasally challenged with the PRRSV-1 07V063 strain at 6 weeks of age. The mean viral titer ± standard error of the mean viral titer is shown as error bars for each experimental group.

In the E+ piglets, reduced viral titers were observed in the vaccinated piglets (both E+ IM and E+ ID) compared to the non-vaccinated piglets from 3 dpi until 14 dpi (Figure 8A). At 5 dpi, a significantly lower (*p* = 0.012) viral titer was observed in the E+ IM piglets (2.46 ± 1.10 TCID_50_/mL) compared to the E+ NoVac piglets (3.83 ± 0.50 TCID_50_/mL). In contrast, the viral titers remained similar between the vaccinated and non-vaccinated E− piglets until 7 dpi. At 10 dpi, a clear vaccine effect was observed in the E− piglets, with a significantly lower (*p* = 0.040) viral titer in the E− IM piglets (3.35 ± 0.70 TCID_50_/mL) and a non-significantly lower (*p* = 0.080) viral titer in the E− ID piglets (3.33 ± 0.85 TCID_50_/mL), compared to the E− NoVac group (4.46 ± 0.84 TCID_50_/mL). At 14 dpi, a similar trend was observed, with a non-significantly (*p* = 0.064) lower viral titer in the E− IM piglets (3.07 ± 0.49 TCID_50_/mL) and a significantly lower (*p* = 0.021) viral titer in the E− ID piglets (2.63 ± 1.12 TCID_50_/mL) compared to the E− NoVac piglets (4.11 ± 0.81 TCID_50_/mL).

**Figure 9:**
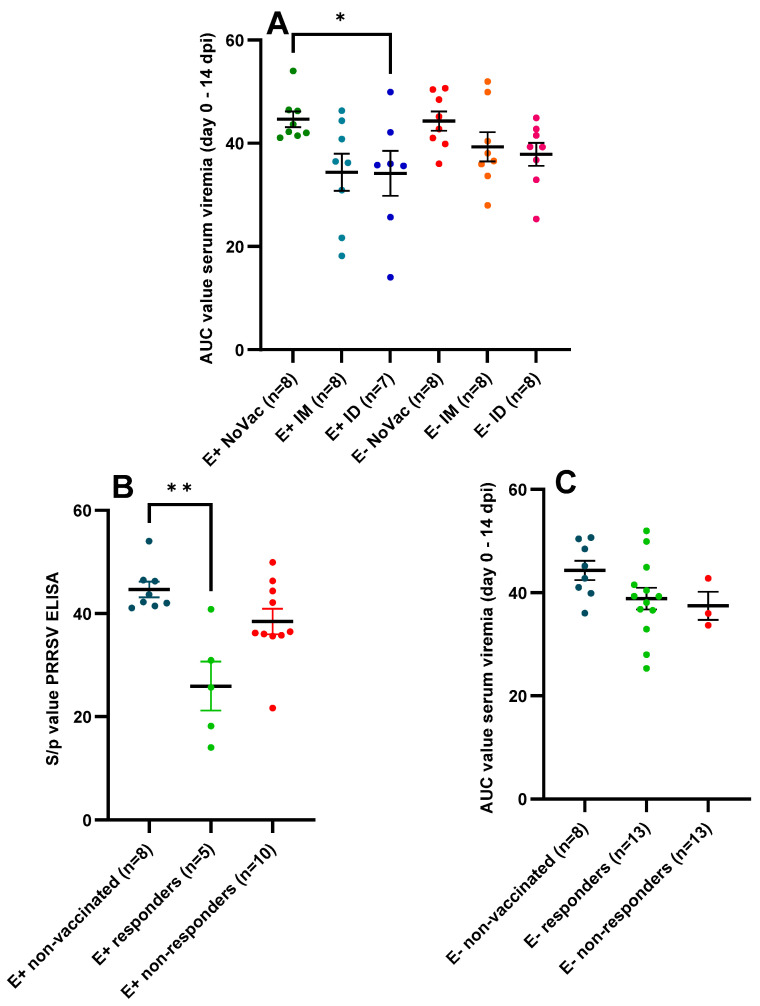
Serum viral load, quantified by in vitro viral titration, in piglets originating from routinely PRRSV vaccinated, seropositive sows (E+ piglets) or piglets originating from routinely PRRSV vaccinated, seronegative/slightly seropositive sows (E− piglets). Piglets were either non-vaccinated (NoVac), intramuscularly vaccinated (IM), or intradermally vaccinated (ID) at 3 weeks of age with the same PRRSV-1 MLV as used in the sows, and all piglets were intranasally challenged with the PRRSV-1 07V063 strain at 6 weeks of age. The mean AUC value ± standard error of the mean AUC value are shown as error bars. (**A**) AUC values for the E+ NoVac, E+ IM, E+ ID, E− NoVac, E− IM, and E− ID piglets. (**B**) AUC values for the E+ subgroups based on the vaccine antibody response. E+ responder piglets had a vaccine antibody response by 3 weeks post-vaccination, while the E+ non-responder piglets lacked a vaccine antibody response by 3 weeks post-vaccination. (**C**) AUC values for the E− subgroups based on the vaccine antibody response at 3 weeks post-vaccination.

The comparison of the AUC values based on the viral titration (day 0 until 14 dpi) confirmed the positive effect of the PRRSV-1 vaccination on the viral load post-challenge. The E+ NoVac piglets (44.65 ± 4.31) had a significantly higher (*p* = 0.049) AUC-value compared to the E+ ID piglets (34.16 ± 11.52) and a non-significantly higher (*p* = 0.068) AUC-value compared to the E+ IM piglets (34.38 ± 10.20) (Figure 9A). Additionally, a non-significantly reduced viral load was observed in the E− IM (39.32 ± 8.05) and E− ID (37.85 ± 6.26) piglets compared to the E− NoVac (44.30 ± 5.32) piglets. The AUC-values in the five E+ responding piglets (25.92 ± 10.60) were significantly lower (*p* = 0.0044) than the AUC-values in the eight E+ NoVac piglets (44.65 ± 4.31) and numerically lower (*p* = 0.16) than the AUC values in the ten E+ non-responding piglets (38.45 ± 7.84) (Figure 9B). Finally, no significant differences were observed between the AUC values of the E− NoVac piglets (44.30 ± 5.32), E− responder piglets (38.85 ± 7.57), and E− non-responder piglets (37.45 ± 4.73).

### 3.6. Antibody Kinetics Post-Challenge

The overall kinetics of antibody induction post-challenge were relatively similar between the vaccinated and non-vaccinated piglets (Supplementary Figure S3). All E+ NoVac piglets seroconverted between 7 dpi and 10 dpi. Additionally, the E+ vaccinated piglets that initially lacked a vaccine antibody response (seronegative at 3 wpv) did have a challenging antibody response by 10 dpi. Six out of eight E− NoVac piglets seroconverted between 7 dpi and 10 dpi; the remaining E− NoVac piglets had a delayed antibody response and seroconverted from 14 dpi onwards. Furthermore, four of the E− NoVac piglets had a low antibody titer throughout the study. The three non-responding E− piglets seroconverted by 10 dpi as well. 

**Figure 10:**
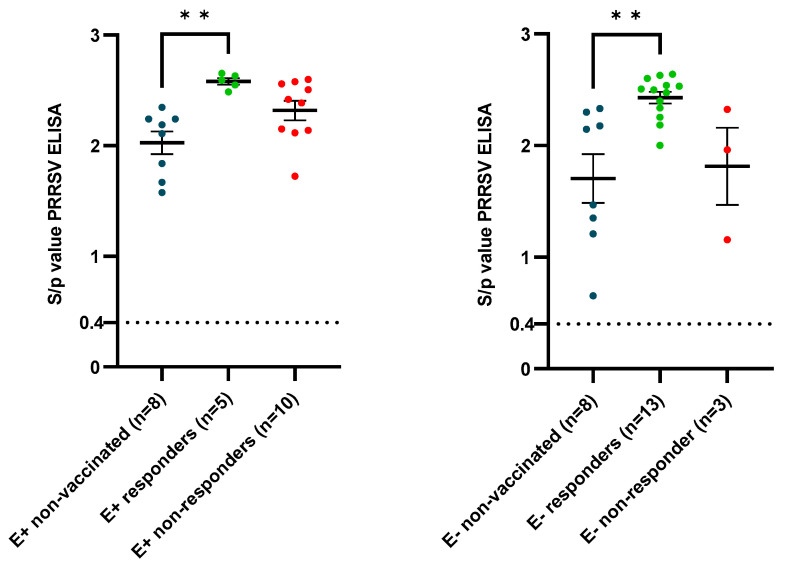
PRRSV-specific antibodies at 41 days post-infection originating from routinely PRRSV vaccinated, seropositive sows (E+ piglets; **left**) or piglets originating from routinely PRRSV vaccinated, seronegative/slightly seropositive sows (E− piglets; **right**)). Piglets were either vaccinated or non-vaccinated (NoVac) at 3 weeks of age with the same PRRSV-1 MLV as used in the sows, and all piglets were intranasally challenged with the PRRSV-1 07V063 strain at 6 weeks of age. Subgroups were made in the vaccinated piglets, based on the vaccine antibody response at 3 weeks post-vaccination, with responders having a vaccine antibody response and non-responders lacking a vaccine antibody response. The mean S/*p* value ± standard error of the mean is shown as error bars for each experimental group. A dotted line represents the cut-off value for seropositivity.

The successful PRRSV-1 vaccination, evidenced by a vaccine antibody response at 3 wpv, had a priming effect on the elicited serological response by the end of the study (41 dpi), both in the E+ and E− piglets (Figure 10). A significantly higher (*p* = 0.0031) and numerically higher (*p* = 0.17) antibody titer was observed in the E+ responders (2.58 ± 0.067) compared to the E+ NoVac piglets (2.03 ± 0.29) and E+ non-responders (2.32 ± 0.28), respectively. Additionally, a significantly higher (*p* = 0.0031) and numerically higher (0.086) antibody titer was observed in the E− responders (2.43 ± 0.19) compared to the E− NoVac piglets (1.70 ± 0.62) and E− non-responders (1.81 ± 0.60), respectively. 

### 3.7. Induction of Interferon-γ Secreting Cells

**Figure 11:**
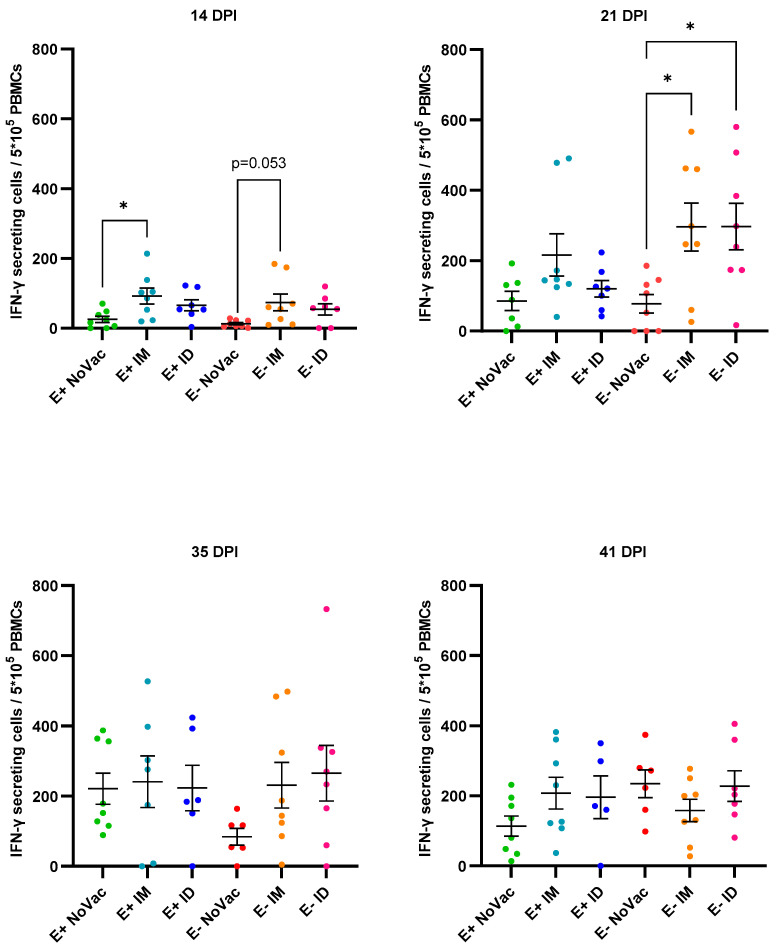
Overview of interferon-γ secreting cells (IFN-γ SCs) specific for the PRRSV-1 DV strain in piglets originating from routinely PRRSV vaccinated, seropositive sows (E+ piglets) or piglets originating from routinely PRRSV vaccinated, seronegative/slightly seropositive sows (E− piglets) Piglets were either non-vaccinated (NoVac), intramuscularly vaccinated (IM) or intradermally vaccinated (ID) at 3 weeks of age with the same PRRSV-1 MLV as used in the sows. All piglets were intranasally challenged with the PRRSV-1 07V063 strain at 6 weeks of age. The number of PRRSV-specific IFN-γ SCs was quantified at 14, 21, 35, and 41 days post-infection (dpi). The mean number of IFN-γ SCs ± standard error of the mean are shown as error bars for each experimental group.

The PRRSV-1 vaccination had a positive effect on the early induction of PRRSV-specific IFN-γ secreting cells (IFN-γ SCs) post-challenge (Figure 11). At 14 dpi, a significantly higher (*p* = 0.028) number of IFN-γ SCs was observed in the E+ IM piglets (92.44 ± 64.10) compared to the E+ NoVac piglets (25.50 ± 25.25), while the number of IFN-γ SCs was numerically higher in the E+ ID piglets compared to the E+ NoVac piglets. At the same time point, a non-significant higher number of IFN-γ SCs was observed in the E− vaccinated piglets compared to the E− NoVac piglets. At 21 dpi, the number of IFN-γ secreting cells remained numerically higher in the vaccinated E+ piglets compared to the non-vaccinated E+ piglets. Moreover, the number of IFN-γ SCs was significantly higher in both the E− IM piglets (296.1 ± 193.2; *p* = 0.031) and E− ID piglets (296.8 ± 186.8; *p* = 0.017) compared to the E− NoVac piglets (77.75 ± 74.42). Finally, the observed differences in IFN-y-secreting cells between the non-vaccinated and vaccinated piglets became less pronounced by the end of the study.

### 3.8. Cytokine Induction in Serum

**Figure 12:**
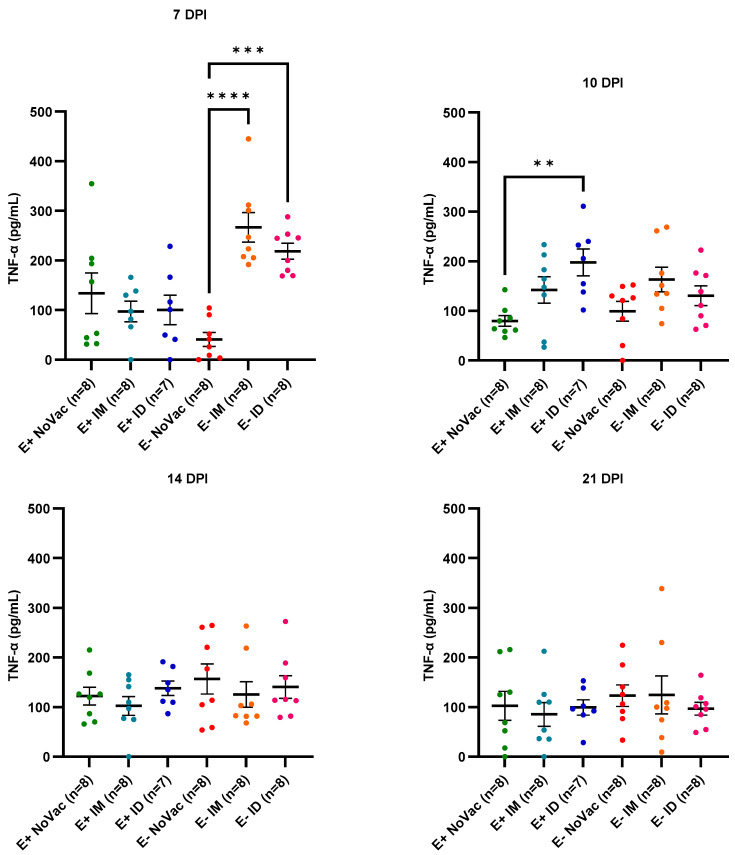
Evolution of serum TNF-α in piglets originating from routinely PRRSV vaccinated, seropositive sows (E+ piglets) or piglets originating from routinely PRRSV vaccinated, seronegative/slightly seropositive sows (E− piglets). Piglets were either non-vaccinated (NoVac), intramuscularly vaccinated (IM), or intradermally vaccinated (ID) at 3 weeks of age with the same PRRSV-1 MLV as used in the sows, and all piglets were intranasally challenged with the PRRSV-1 07V063 strain at 6 weeks of age. The serum concentration of TNF-α was determined at 7, 10, 14, and 21 days post-infection (DPI) using a commercial ELISA assay. Results are shown as dots for each piglet, with error bars representing the mean concentration of serum TNF-α ± standard error of the mean concentration of serum TNF-α for each experimental group.

An overall poor induction of serum IFN-γ was observed in all experimental groups during the first days post-challenge (Supplementary Figure S4). In contrast, a high TNF-α induction was observed from 7 dpi until 21 dpi, with a higher early induction in the vaccinated piglets compared to the non-vaccinated piglets (Figure 12). At 7 dpi, a significantly higher TNF-α induction was observed in the serum of both the E− IM piglets (266.7 ± 84.68 pg/mL; *p* < 0.0001) and E− ID piglets (218.7 ± 44.91 pg/mL; *p* = 0.0001) compared to the serum TNF-α concentration of the E− NoVac piglets (40.88 ± 39.50 pg/mL). At 10 dpi, a significantly higher (*p* = 0.0026) TNF-α induction was observed in the E+ ID piglets (197.8 ± 71.30 pg/mL) compared to the E+ NoVac piglets (79.90 ± 30.67 pg/mL). At 14 and 21 dpi, the TNF-α concentrations of all experimental groups were at a similar level.

## 4. Discussion

Vaccination against the Porcine Reproductive and Respiratory Syndrome Virus (PRRSV) is the main tool, together with adequate biosecurity, to limit the potential losses caused by this small RNA virus [10]. Unfortunately, the effectiveness of PRRSV vaccination is suboptimal, mainly due to the large genetic diversity of the virus and its capability to modulate the immune response [14,15,16]. Additionally, several studies reported the unsuccessful PRRSV vaccination of both piglets and sows, resulting in seronegative animals despite vaccination [26,27,28,29,30,31,32]. Our research group aimed to unravel the role of these non-responding animals by assessing their prevalence, origin, and possible consequences [26,27,33].

### 4.1. Prevalence and Consequences of Multiple Vaccinated, Seronegative Sows

The presence of sows lacking an adequate PRRSV antibody response despite repeated immunization with a modified live vaccine (MLV) has been reported as early as 1999 by Baker et al. but remained understudied [31]. In a recent field study, our research group assessed the prevalence of these non-responding sows by sampling 1400 sows originating from seventy PRRSV vaccinating sow herds in Belgium [26]. The overall prevalence of the multiple vaccinated but seronegative sows was relatively low, ranging from 3.5% to 4.1%. However, in 40% of the sampled herds, at least one non-responding sow was present on the twenty sows sampled. In the current study, a screening was performed in 56 PRRSV-vaccinated sows at 90 days of gestation (one month after the last PRRSV MLV vaccination). Five out of the 56 sampled sows could be considered as non-responders to the routine vaccination, testing seronegative or very slightly seropositive (S/*p* values around the cut-off for seropositivity).

The true origin of the non-responsiveness remains unknown for now. Interestingly, the combination strategy of vaccination with both an MLV and an inactivated vaccine (IV) as a booster had a clear beneficial effect on reducing the likelihood of having seronegative sows [26]. This suggests that the theory of sows showing energy towards the used MLV strain, as proposed by Baker et al., is quite likely [31]. Furthermore, other studies showed that this vaccination strategy (MLV + IV) has a beneficial effect, both on sustaining the immune response and on the outcomes in endemically infected farms [37,38]. 

The possible consequences of the non-responding sows can be divided into two large categories, namely, the consequences for the sow and the consequences for the progeny. Firstly, the lack of ELISA antibodies and the significantly lower amount of neutralizing antibodies observed in the non-responding sows suggest that they might be less protected against a field infection [26]. To confirm this hypothesis, an experimental trial should be conducted in which both vaccinated seropositive and vaccinated seronegative sows are challenged. Alternatively, an in-depth investigation into the cell-mediated immunity (CMI) of both groups of sows could aid in predicting their outcome after the challenge. Secondly, it was shown in both the current study and in our previous studies that piglets born from the non-responding sows lack the presence of PRRS-specific maternally-derived antibodies (MDAs) [27,33]. Given the immature immune system of neonatal pigs, the transfer of maternal immunity is needed to protect piglets during the immune maturation process [39]. Consequently, it could be hypothesized that piglets born without MDAs are less protected against a field infection in the early stages of life compared to piglets with MDAs. To investigate this, our research group conducted a challenging experiment including piglets born from both PRRSV-vaccinated seropositive sows (E+ piglets, with MDAs) and piglets born from PRRSV-vaccinated seronegative sows (E− piglets, without MDAs) [33]. After the experimental challenge at 4 weeks of age (woa), the E− piglets had a higher serum viral load and nasal shedding in the first days post-infection (dpi) compared to the E+ piglets. Additionally, higher fever induction was observed in the E− piglets throughout this study. These results suggest that the subpopulation of piglets without PRRSV-specific MDAs, which is present in a large proportion of the PRRSV-vaccinating sow herds, might play a role in the early stages of a PRRSV outbreak or in the enhanced transmission in endemically infected herds.

### 4.2. Interference of MDAs on the PRRSV Piglet Vaccine Response

The presence of vaccinated piglets lacking an adequate PRRSV antibody response is related to the interference of MDAs, a phenomenon that plays a role in a whole range of both human and veterinary diseases. Several mechanisms of inhibition of seroconversion due to the presence of maternal immunity have been described. The best-characterized form of MDA interference involves the presence of neutralizing MDAs. The interference of these MDAs is straightforward: neutralization of the administered MLV inhibits the humoral response. Alternatively, MDAs, both neutralizing and non-neutralizing, can inhibit the vaccine response by binding to the presented MLV antigens on the cell surface of infected cells, resulting in the cell-mediated or complement-mediated clearance of the infected cells. Moreover, MDAs can bind to circulating MLV particles in the blood, leading to the masking of immunogenic epitopes or to opsonization and subsequent phagocytic clearance of the MLV particles. Finally, MDAs can cross-link with B-cell receptors, resulting in suppression of the B cell function, or they can interact with B cells in the germinal center, influencing the B cell differentiation [40,41]. In pigs, the inhibition of vaccination due to the presence of MDAs has been described for several diseases, including swine influenza and classical swine fever [42,43,44]. In the case of homologous PRRSV vaccination (same MLV used in piglets as in sows), a clear interference of both neutralizing and non-neutralizing MDAs on the vaccine response has been described in different studies [27,28,29]. Conversely, in the study of Kraft et al., the homologous PRRSV vaccination of piglets at both 2 woa and 3 woa was successful, even in the presence of MDAs [45]. It could be hypothesized that the lack of MDA interference in this study is related to either the genetic background of the pigs or to the MLV strain. Additionally, Balasch et al. described the capability of an MLV to overcome maternal immunity when this MLV is administered to 1-day-old piglets originating from sows that have been vaccinated once [46]. Finally, in a recent study by Aguire et al., the use of a heterologous PRRSV vaccine was able to overcome the MDA interference [47]. Although the use of a heterologous PRRSV vaccine in piglets is beneficial in overcoming the MDA interference, it is not recommended to use different MLV strains in the same herd, given the risk for recombination events [19,20,21].

### 4.3. Vaccine Effectiveness after Challenge in Piglets Born from Responding and Non-Responding Sows

In the current study, our research group aimed to further elucidate the consequence of the multivaccinated seronegative sows for the progeny by comparing the vaccine effectiveness after experimental challenge in piglets born from both responding sows (E+ piglets) and piglets born from non-responding sows (E− piglets). E+ piglets and E− piglets were homologous PRRSV vaccinated, either intramuscular (IM) or intradermal (ID), at 3 woa, followed by an experimental PRRSV-1 challenge at 6 woa. A clear MDA interference was observed in the E+ piglets, with 10/15 (66.7%) of the vaccinated E+ piglets lacking a vaccine antibody response by 3 wpv. In contrast, only 3/16 (18.8%) of the E− vaccinated piglets lacked a vaccine-induced antibody response by 3 wpv. The presence of MDA interference in the E+ vaccinated piglets corroborates with our previous study and with the study of Renson et al. (2019), in which 45% (at 8 wpv) and 56% (at 5 wpv) of the piglets with MDAs lacked an antibody response, respectively [27,29]. In both studies, the same PRRSV-1 MLV was used as in the current study. More research is warranted to understand why the MDA interference only occurs in a proportion of the piglets and which immunological mechanisms are responsible for the MDA interference after PRRSV vaccination. In the current study, none of the piglets had neutralizing MDAs at the moment of vaccination, suggesting that non-neutralizing MDAs are capable of interfering with the PRRSV MLV response as well. However, next to MDAs, the presence of high serum IFN-α has been shown to inhibit the replication of PRRSV MLVs [48]. In the study of Renson et al. (2019), PRRSV MLV viremia was only observed in 12.5% of vaccinated piglets with low neutralizing MDA titers at 1 wpv. This was in sharp contrast with a previous study conducted by this research group, in which MLV viremia was observed in 60% of vaccinated piglets with low neutralizing MDA titers at 2 wpv [28]. A comparison of the IFN-α levels prior to vaccination revealed that the IFN-α concentration was significantly higher in their second study [29] compared to their first study [28]. Consequently, the authors suggested that next to the interference of neutralizing MDAs, a high concentration of serum IFN-α can contribute to the inhibition of the vaccine response [29]. In the current study, serum IFN-α was not investigated, and thus, the possible inhibiting effect of serum IFN-α on the observed lack of vaccine response cannot be confirmed nor ruled out.

The MLV administration route, IM or ID, did not influence the number of piglets without a vaccine antibody response. Additionally, at 3 wpv, both routes of vaccination induced a limited number of PRRSV-specific IFN-γ secreting cells. However, the number of IFN-γ secreting cells was numerically higher in the ID-vaccinated piglets compared to the IM-vaccinated piglets. Post-challenge, the vaccine effectiveness was similar between IM and ID routes, with a similar reduction in viremia and similar induction of CMI responses. These results are in accordance with the recent study of Renson et al. (2024), in which the systemic and mucosal immune responses and vaccine efficacy were compared between IM and ID vaccinated piglets using the same PRRSV MLV as used in the current study. In this study, the ID-vaccinated piglets showed an earlier induction of CMI responses post-vaccination. However, the immune responses and viremia were similar between IM and ID-vaccinated piglets post-challenge [49].

Vaccination of both E+ and E− piglets induced a limited reduction in viral load compared to the non-vaccinated piglets. This limited vaccine effectiveness can partially be explained by the time of challenge. In this study, the piglets were challenged at an early time-point, 3 wpv, which is one week earlier than the onset of immunity indicated on the MLV leaflet. Additionally, a clear influence of vaccine non-responsiveness on the reduction in viral load was observed in the E+ vaccinated piglets. A significantly lower viremia was observed in the five responding E+ piglets compared to the eight non-vaccinated piglets, while a trend towards lower viremia was observed in the responding E+ piglets compared to the non-responding E+ piglets. Additionally, the ten non-responding piglets had a similar viremia compared to the eight non-vaccinated E+ piglets. These results suggest that piglets lacking a vaccine response due to the presence of MDAs are less protected against infection with a field isolate, which is in accordance with the results of Renson et al. (2019). This has important implications for field conditions since quite a high proportion of piglets will not be protected when homologously vaccinated at 3 woa. Alternative vaccine strategies, such as a double vaccination of piglets (early age + later in the nursery), could potentially ensure that all piglets have a sufficiently high PRRSV immune status until the end of the fattening period. In the vaccinated E− piglets, a delayed vaccine effect was observed, with a reduced viral load from 7 dpi to 14 dpi, compared to the non-vaccinated E− piglets. This delay in vaccine effectiveness could be explained by the early time-point of challenge. Surprisingly, the three non-responding E− piglets had a significantly lower overall viremia compared to the eight non-vaccinated E− piglets, while the reduction in overall viremia was not significant in the thirteen responding E− piglets compared to the non-vaccinated E− piglets. It could be hypothesized that a high, sustained serum IFN-α concentration in these piglets might be responsible for the lack of vaccine response and the lower viremia post-challenge. Alternatively, a certain resistant phenotype in these non-responding E− piglets might influence both the vaccine response and the outcome of infection.

Finally, both the E+ and E− non-responding piglets had a lower antibody titer compared to the responding piglets by the end of the study, suggesting the absence of immune priming in these non-responders. Moreover, some additional differences were observed between the challenge outcomes in the E+ piglets and E− piglets. First, a non-significant, higher fever induction was observed in the E− piglets (both vaccinated and non-vaccinated) compared to the E+ piglets, which is in line with the findings of our previous experimental study [33]. Additionally, early and intense induction of both serum TNF-α (at 10 dpi) and IFN-γ secreting cells (at 21 dpi) was observed in the E− vaccinated piglets compared to the E− non-vaccinated piglets, while this difference was less pronounced between the E+ vaccinated and E+ non-vaccinated piglets. By the end of the study, at 41 dpi, a relatively low antibody titer was observed in four E− non-vaccinated piglets and in one E− IM non-responding piglet. Finally, a delayed and less pronounced vaccine effect on the serum viral load was observed in the E− vaccinated piglets in comparison to the vaccine effect in the E+ vaccinated piglets. Further research is warranted to find an explanation for these abovementioned differences. Given the fact that the E− piglets originate from sows that have an inadequate response to the routine MLV vaccination, some genetic factors might be involved.

## 5. Conclusions

The current study identified differences in vaccine effectiveness in piglets born from routinely PRRSV-vaccinated, seropositive (responding), and seronegative (non-responding) sows. Additionally, the study confirmed the relevance of MDA interference in PRRSV-vaccinated piglets. Vaccinated piglets lacking an adequate antibody response to the PRRSV MLV vaccination due to the presence of MDAs were less protected against an experimental challenge compared to vaccinated piglets that had an adequate antibody response in the presence of MDAs. This is of importance in field conditions since the MDA interference can be quite high, with up to 66.7% of the vaccinated piglets lacking an adequate antibody response at 3 weeks post-vaccination when homologously vaccinated at 3 weeks of age. Alternative vaccine strategies should be investigated to ensure that all vaccinated piglets have a sufficient PRRSV immune status until the end of the production cycle. Conversely, piglets born from non-responding sows lacking a vaccine-induced antibody response seem to be more resistant to experimental challenge. Given their mother’s irregular immune response to routine PRRSV vaccination, certain genetic factors might play a role in this resistant phenotype. Some additional differences in the challenge outcomes between piglets born from PRRSV-vaccinated, seropositive, and PRRSV-vaccinated, seronegative sows were observed, which warrants further investigation.

## Figures and Tables

**Table 1:** Overview of the different experimental groups. E+ piglets were born from three PRRSV-vaccinated, responding sows. E− piglets were born from three PRRSV-vaccinated, non-responding sows. All piglets were intranasally challenged at 6 weeks of age (woa) with 2 mL containing 10^5.5^ TCID_50_/mL of the PRRSV-1 07V063 strain.

Experimental Group (*n*)	Vaccination (Age)	Compartment
E+ NoVac (*8*)	No vaccination	1
E+ IM (*8*)	Porcilis IM (3 woa)	2
E+ ID (*8*) *	Porcilis ID (3 woa)	3
E− NoVac (*8*)	No vaccination	4
E− IM (*8*)	Porcilis IM (3 woa)	5
E− ID (*8*)	Porcilis ID (3 woa)	6

* One E+ ID piglet died during blood sampling at 5 dpi and was excluded from analysis.

**Table 2:** Overview of the PRRSV serological status of six selected PRRSV-vaccinated sows and the presence of PRRSV-specific maternally-derived antibodies in their progeny. A commercially available ELISA kit was used to detect PRRSV-specific Abs, with results shown as sample-to-positive (S/p) values (cut-off for seropositivity: S/*p* value ≥ 0.4). A virus neutralization assay (VN) was used to determine the presence of neutralizing antibodies against the vaccine strain, with results shown as the Log_2_ VN titer. Sows were intramuscularly PRRSV-vaccinated at 60 days of gestation and 6 days post-farrowing (dpf); sow sampling was performed at 90 days of gestation and 14 dpf; piglet sampling was performed at 14 dpf.

Sow	Parity	S/*p* Value (90 Days Gestation)	VN Titer (90 Days Gestation)	S/*p* Value (14 dpf)	S/*p* Value Selected Piglets (14 dpf) (Mean ± SD)	VN Titer Selected Piglets (14 dpf) (Mean ± SD)
1	3	2.08	0	1.76	1.86 ± 0.10	0 ± 0
2	3	2.16	2	1.95	2.04 ± 0.05	0 ± 0
3	8	2.79	0	2.57	2.17 ± 0.10	0 ± 0
4	3	0.25	0	0.18	0.13 ± 0.02	0 ± 0
5	3	0.43	0	0.52	0.37 ± 0.08	0 ± 0
6	7	0.51	0	0.38	0.41 ± 0.05	0 ± 0

## Data Availability

The data presented in this study are available on request from the corresponding author. The data are not publicly available due to privacy reasons.

## Supplementary Materials

The following supporting information can be downloaded at: https://www.mdpi.com/article/10.3390/vaccines12030257/s1, Figure S1A: PRRSV-specific antibodies in fifty-six PRRSV-vaccinated sows sampled at 90 days of gestation (one month after the last PRRSV MLV vaccination); Figure S1B: PCV2-specific antibodies in the three selected PRRSV seropositive sows and three selected PRRSV seronegative sows; Figure S2: Evolution of body temperature in piglets born from PRRSV vaccinated, seropositive sows (E+ piglets) or PRRSV vaccinated, seronegative/slightly seropositive sows (E− piglets); Figure S3: Evolution of PRRSV-specific antibodies in piglets born from PRRSV vaccinated, seropositive sows (E+ piglets) or PRRSV vaccinated, seronegative/slightly seropositive sows (E− piglets); Figure S4: Evolution of serum IFN-γ in piglets born from PRRSV vaccinated, seropositive sows (E+ piglets) or PRRSV vaccinated, seronegative/slightly seropositive sows (E− piglets).
