# Supplementary material for: PRRSV-Vaccinated, Seronegative Sows and Maternally Derived Antibodies (II): Impact on PRRSV-1 Vaccine Effectiveness and Challenge Outcomes in Piglets
Source: Vaccines (Basel). 2024 Mar 1;12(3):257. doi: 10.3390/vaccines12030257 (PMC10976001; doi:10.3390/vaccines12030257)
Supplement: Supplementary file 1 [file vaccines-12-00257-s001.zip › vaccines-2883453-supplementary Figures.pdf]

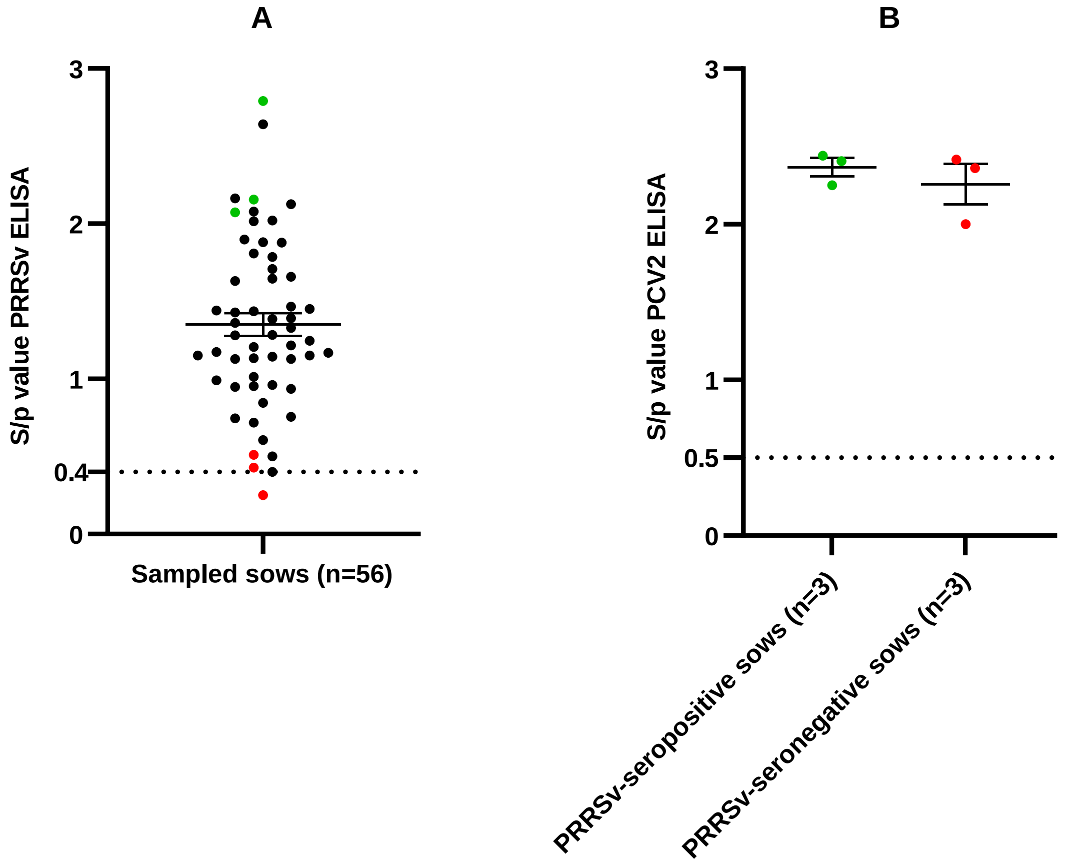

Supplementary Figure S1A. PRRSV-specific antibodies in fifty-six PRRSV-vaccinated sows sampled at 90 days of gestation (one month after the last PRRSV MLV vaccination). Three PRRSV seropositive sows (green dots – responders) and three PRRSV seronegative / slightly seropositive sows (red dots – non-responders) were selected. **Supplementary Figure S1B.** PCV2-specific antibodies in the three selected PRRSV-seropositive sows and three selected PRRSV-seronegative sows. Results are shown as dots representing the individual sample-to-positive (S/p) values for each sow. The cut-off value for seropositivity in each ELISA test is shown as a dotted line. Error bars represent the mean S/p-value ± standard error of the mean (SEM).

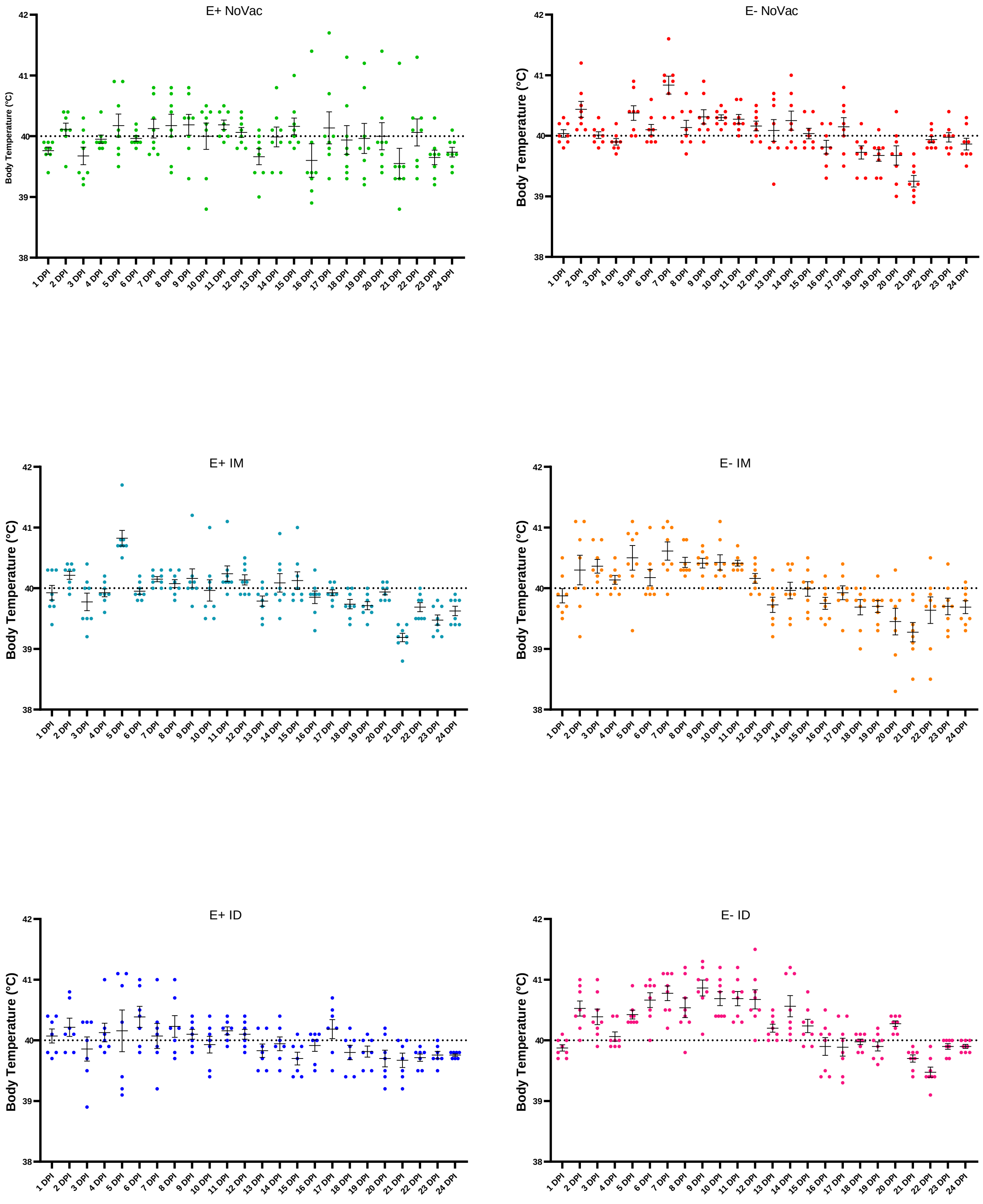

Supplementary Figure S2. Evolution of body temperature in piglets born from PRRSV vaccinated, seropositive sows (E+ piglets) or PRRSV vaccinated, seronegative / slightly seropositive sows (E- piglets). Piglets were either non-vaccinated (NoVac), intramuscularly vaccinated (IM), or intradermally vaccinated (ID) at 3 weeks of age with the same PRRSV-1 MLV as used in the sows. All piglets were intranasally challenged with the PRRSV-1 07V063 strain at 6 weeks of age. Results are shown as dots for each piglet, with error bars representing the mean body temperature ± standard error of the mean body temperature for each experimental group at each time point.

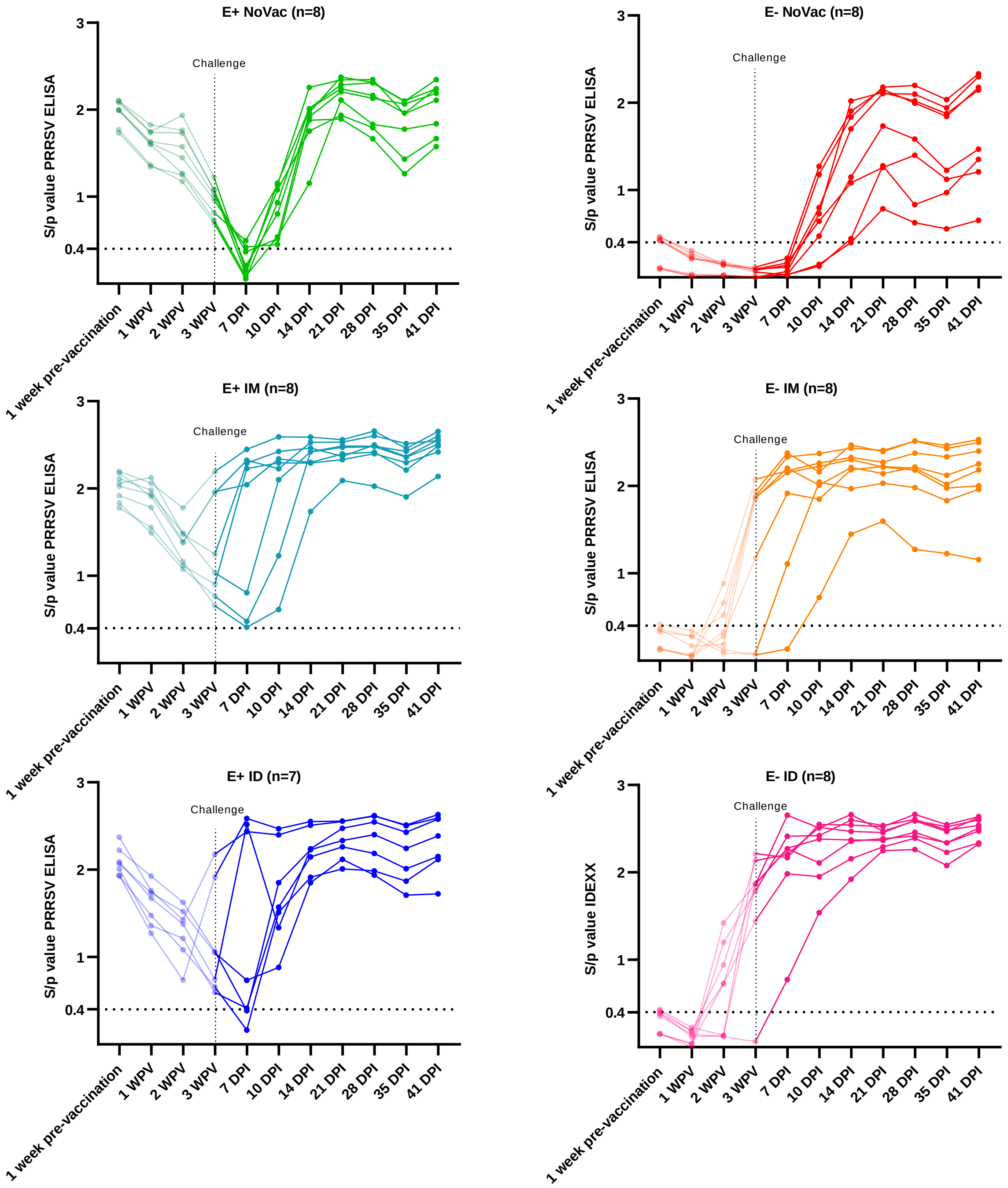

Supplementary Figure S3. Evolution of PRRSV-specific antibodies in piglets born from PRRSV vaccinated, seropositive sows (E+ piglets) or PRRSV vaccinated, seronegative / slightly seropositive sows (E- piglets). Piglets were either non-vaccinated (NoVac), intramuscularly vaccinated (IM) or intradermally vaccinated (ID) at 3 weeks of age with the same PRRSV-1 MLV as used in the sows. All piglets were intranasally challenged with the PRRSV-1 07V063 strain at 6 weeks of age. Sample-to-positive values (S/p values) are shown as dots for each piglet. The cut-off value for seropositivity (S/p-value ≥ 0.4) is indicated with a dotted line.

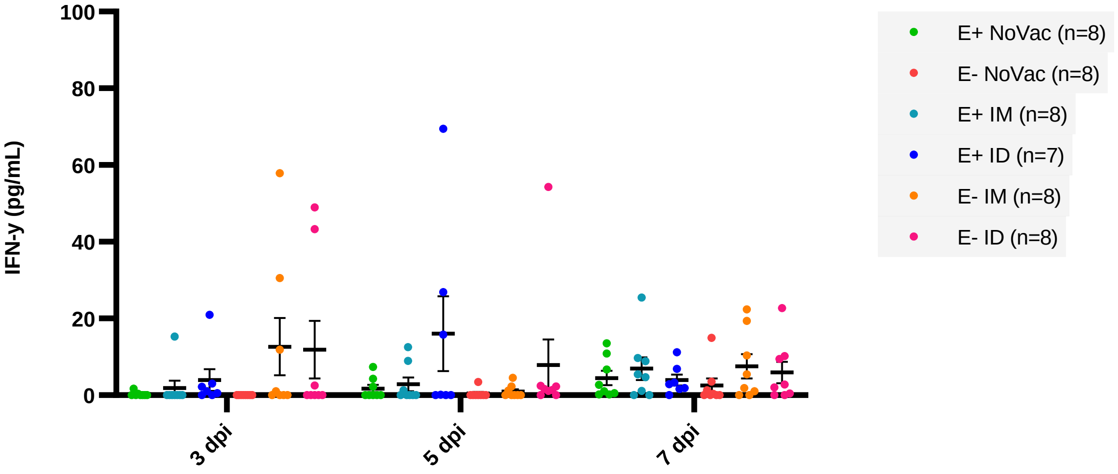

Supplementary Figure S4. Evolution of serum IFN-γ in piglets born from PRRSV vaccinated, seropositive sows (E+ piglets) or PRRSV vaccinated, seronegative / slightly seropositive sows (E- piglets). Piglets were either non-vaccinated (NoVac), intramuscularly vaccinated (IM) or intradermally vaccinated (ID) at 3 weeks of age with the same PRRSV-1 MLV as used in the sows. All piglets were intranasally challenged with the PRRSV-1 07V063 strain at 6 weeks of age. The serum concentration of IFN-γ was determined at 3, 5 and 10 days post-infection (DPI) using a commercial ELISA assay. Results are shown as dots for each piglet, with error bars representing the mean concentration of serum IFN-γ ± standard error of the mean concentration of serum IFN-γ for each experimental group at each time point.
